# Supplementary material for: New Caregiver Diagnoses of Severe Depression and Child Asthma Controller Medication Adherence
Source: Int J Environ Res Public Health. 2023 May 29;20(11):5986. doi: 10.3390/ijerph20115986 (PMC10252375; doi:10.3390/ijerph20115986)
Supplement: Supplementary file 1 [file ijerph-20-05986-s001.zip › 6.2 ijerph-2217207 supplementary updated 6.2.pdf]

Supplementary Materials: Caregiver New Diagnoses of Severe Depression and Child Asthma Controller Medication Compliance

Table S1. Summary of Several Previous Studies of the Relationship Between Caregiver Depression and Children's Asthma and/or Medication Compliance.

Study	Design	Sample Size	Results
Bartlett et al., 2004 [12]	A cross-sectional study examining correlations between number of maternal depressive symptoms and mother reported child medication adherence.	177 minority inner city children with asthma.	Mothers with more depressive symptoms reported more problems with medication adherence and less confidence in the ability of medications to control the child's asthma.
Brew et al. 2018 [10]	Administrative health records were linked for mothers and children to identify periods of maternal depression.	All children born in Sweden from July 2006 to December 2009 (n = 360 526).	Longer periods of maternal depression increased the risk of childhood asthma.
Brown et al. 2008 [26]	Observational study.	8 women treated for depression and their children.	Both parental depression and children's asthma symptoms improved.
Kozyrskyi et al., 2008 [11]	A birth cohort study following children from birth to age seven in administrative health records and using logistic regression to control for asthma risk factors.	A 1995 Manitoba, Canada birth cohort of 13,907 children.	Independent of well-known asthma risk factors, there was an increased risk of childhood asthma (OR, 1.25; 95% CI, 1.01-1.55) among children exposed to continued maternal depression and anxiety from birth to age 7.
Leão et al, 2009 [4]	Comparison of mothers of children with and without asthma in a pediatric outpatient clinic in Brazil.	80 mothers of children with asthma and 160 mothers of children without asthma.	The prevalence of depression was higher among mothers of asthmatic children compared with mothers of non-asthmatic children (43.8% vs. 17.5%, $p <$ 0.001)

Margolis et al., 2020 [14]	A longitudinal study following mothers and children for XX.	204 inner-city children recruited after emergency room visits for asthma.	Increases in self-reported maternal depression were associated with reductions in self-reported medication compliance. Changes in a caregiver's depression status were more important than baseline levels.
Otsuki et al., 2010 [15]	Phone surveys conducted 6 months apart.	262 African American mothers of children with asthma.	Time 1 maternal depressive symptoms predicted Time 2 child asthma symptoms but the reverse was not true.
Rodriquez et al., 2017 [27]	Survey of parents regarding their depressive symptoms and their child's asthma control.	78 parents (90% female).	Parental depressive symptoms were negatively related to children's asthma control.

Table S2. List of Asthma Diagnosis Codes

CODE	Description
J45	Asthma
J452	Mild intermittent asthma
J4520	Mild intermittent asthma, uncomplicated
J4521	Mild intermittent asthma with (acute) exacerbation
J4522	Mild intermittent asthma with status asthmaticus
J453	Mild persistent asthma
J4530	Mild persistent asthma, uncomplicated
J4531	Mild persistent asthma with (acute) exacerbation
J4532	Mild persistent asthma with status asthmaticus
J454	Moderate persistent asthma
J4540	Moderate persistent asthma, uncomplicated
J4541	Moderate persistent asthma with (acute) exacerbation
J4542	Moderate persistent asthma with status asthmaticus
J455	Severe persistent asthma
J4550	Severe persistent asthma, uncomplicated
J4551	Severe persistent asthma with (acute) exacerbation
J4552	Severe persistent asthma with status asthmaticus
J459	Other and unspecified asthma

J4590	Unspecified asthma
J45901	Unspecified asthma with (acute) exacerbation
J45902	Unspecified asthma with status asthmaticus
J45909	Unspecified asthma, uncomplicated
J4599	Other asthma
J45990	Exercise induced bronchospasm
J45991	Cough variant asthma
J45998	Other asthma

Table S3. List of the Sample Children's Asthma Medications

Aclidinium Bromide
Aclidinium Bromide-Formoterol Fumarate
Albuterol Sulfate
Aminophylline
Arformoterol Tartrate
Beclomethasone Dipropionate
Beclomethasone Dipropionate HFA
Benralizumab
Budesonide (Inhalation)
Budesonide-Formoterol Fumarate Dihydrate
Budesonide-Glycopyrrolate-Formoterol Fumarate
Ciclesonide
Cromolyn Sodium
Dyphylline-Guaifenesin
Ephedrine HCl
Ephedrine Sulfate
Epinephrine HFA
Flunisolide
Flunisolide HFA
Fluticasone Furoate (Inhalation)
Fluticasone Furoate-Vilanterol
Fluticasone Propionate (Inhalation)
Fluticasone Propionate HFA
Fluticasone-Salmeterol
Fluticasone-Umeclidinium-Vilanterol
Formoterol Fumarate
Glycopyrrolate (Inhalation)
Glycopyrrolate-Formoterol Fumarate
Indacaterol Maleate
Indacaterol Maleate-Glycopyrrolate

Ipratropium Bromide
Ipratropium Bromide HFA
Ipratropium-Albuterol
Isoproterenol HCl
Levalbuterol HCl
Levalbuterol Tartrate
Mepolizumab
Metaproterenol Sulfate
Mometasone Furoate (Inhalation)
Mometasone Furoate-Formoterol Fumarate Dihydrate
Montelukast Sodium
Olodaterol HCl
Omalizumab
Racepinephrine HCl
Reslizumab
Revefenacin
Roflumilast
Salmeterol Xinafoate
Terbutaline Sulfate
Theophylline
Theophylline in Dextrose
Tiotropium Bromide Monohydrate
Tiotropium Bromide-Olodaterol HCl
Umeclidinium Bromide
Umeclidinium-Vilanterol
Zafirlukast
Zileuton

Table S4. Child Fixed Effects Models of the Effects of a Caregiver's New Diagnosis of Severe Depression on Medication Compliance for Children with 30+ Day Supply

	Coefficient estimate	Standard error	P-value	Lower CL	Upper CL
New Depression Diagnosis	-1.753	0.786	0.026	-3.042	-0.464
Full Coverage Previous Quarter	14.112	0.088	0.000	13.967	14.258
Child Age	-3.445	0.017	0.000	-3.473	-3.418
Sibling Count	-1.546	0.114	0.000	-1.732	-1.359
Zip code is Urban	-2.411	1.125	0.032	-4.256	-0.565
Components of SVI					
Poverty Rate	0.094	0.057	0.100	0.000	0.188
Unemployment Rate	-0.145	0.103	0.161	-0.314	0.025

Per Capita Income, 1000s	0.000	0.000	0.021	0.000	0.000
Percent Without High School Diploma	0.243	0.087	0.005	0.100	0.385
Percent Population Age.65 or Older	-0.081	0.062	0.193	-0.182	0.021
Percent Population Age 17 or Younger	0.031	0.083	0.707	-0.105	0.167
Percent Disabled	0.101	0.110	0.359	-0.079	0.281
Percent Single-Parent Households	0.058	0.100	0.564	-0.106	0.222
Percent Minority	-0.002	0.024	0.928	-0.041	0.037
Percent Not Fluent in English	-0.100	0.110	0.360	-0.280	0.080
Percent in Housing with >10 Units	-0.008	0.027	0.768	-0.053	0.037
Percent in Mobile Homes	0.011	0.040	0.777	-0.055	0.078
Percent Crowded Dwellings	-0.231	0.191	0.228	-0.545	0.083
Percent Households with No Vehicle	-0.102	0.070	0.146	-0.217	0.013
Percent in Group Quarters	0.059	0.063	0.347	-0.044	0.162
Percent Uninsured	-0.149	0.082	0.068	-0.283	-0.015

Notes: There are 1,101,657 quarters of child data, or approximately 7.05 quarters per child. Coefficient estimates are from a child fixed effects model that also included all of the variables shown in the table. Confidence limits (CL) are for a 95% confidence interval. This model is similar to Table 2 except that it includes the components of SVI.

1
